# Characterizing the gut phageome and phage-borne antimicrobial resistance genes in pigs

**DOI:** 10.1186/s40168-024-01818-9

**Published:** 2024-06-05

**Authors:** Jun Hu, Jianwei Chen, Yangfan Nie, Changhao Zhou, Qiliang Hou, Xianghua Yan

**Affiliations:** 1 National Key Laboratory of Agricultural Microbiology, Hubei Hongshan Laboratory, Frontiers Science Center for Animal Breeding and Sustainable Production, College of Animal Sciences and Technology, Huazhong Agricultural University, Wuhan, Hubei 430070 China; 2 College of Animal Sciences, Fujian Agriculture and Forestry University, Fuzhou, Fujian 350002 China; 3 The Cooperative Innovation Center for Sustainable Pig Production, Wuhan, Hubei 430070 China; 4 Hubei Provincial Engineering Laboratory for Pig Precision Feeding and Feed Safety Technology, Wuhan, Hubei 430070 China; 5 BGI Research, Qingdao, Shandong 266555 China; 6 Laboratory of Genomics and Molecular Biomedicine, Department of Biology, University of Copenhagen, Copenhagen, 2100 Denmark

**Keywords:** Pig, Metagenomic, Gut phageome, PhaBOX, Antimicrobial resistance genes

## Abstract

**Background:**

Mammalian intestine harbors a mass of phages that play important roles in maintaining gut microbial ecosystem and host health. Pig has become a common model for biomedical research and provides a large amount of meat for human consumption. However, the knowledge of gut phages in pigs is still limited.

**Results:**

Here, we investigated the gut phageome in 112 pigs from seven pig breeds using PhaBOX strategy based on the metagenomic data. A total of 174,897 non-redundant gut phage genomes were assembled from 112 metagenomes. A total of 33,487 gut phage genomes were classified and these phages mainly belonged to phage families such as *Ackermannviridae*, *Straboviridae*, *Peduoviridae*, *Zierdtviridae*, *Drexlerviridae*, and *Herelleviridae*. The gut phages in seven pig breeds exhibited distinct communities and the gut phage communities changed with the age of pig. These gut phages were predicted to infect a broad range of 212 genera of prokaryotes, such as *Candidatus *Hamiltonella, *Mycoplasma*, *Colwellia*, and *Lactobacillus*. The data indicated that broad KEGG and CAZy functions were also enriched in gut phages of pigs. The gut phages also carried the antimicrobial resistance genes (ARGs) and the most abundant antimicrobial resistance genotype was diaminopyrimidine resistance.

**Conclusions:**

Our research delineates a landscape for gut phages in seven pig breeds and reveals that gut phages serve as a key reservoir of ARGs in pigs.

Video Abstract

**Supplementary Information:**

The online version contains supplementary material available at 10.1186/s40168-024-01818-9.

## Background

Phages are the most abundant biological entities in earth with an estimated amount of 10^31^ particles [1]. In addition to marine environment and soil, phages are also abundant in mammalian intestine [2] and account for approximately 97.9% of total viruses [3]. Phages have key roles in modulating the gut microbial ecosystem via phage predation [4], lysogeny [5], and horizontal gene transfer [6]. Phages can also contribute to stimulating the mammalian immune response [7–9]. Growing evidence has suggested the application of phages in anti-infective therapy [10], recombinant antibody development [11], and food industry biocontrol [12]. Accumulating studies have focused on the pig gut microbiome because pig provides meat for human consumption and is also an important biomedical model [13, 14]. However, analyses of compositions and functions of gut phages in pigs using large-scale metagenomic data remain limited.

Antimicrobial resistance has become a growing threat to public health [15, 16]. A growing body of studies have shown that besides bacteria, phages also carry antimicrobial resistance genes (ARGs) in several environments, such as fresh water [17], marine habitats [18], raw sewage [19, 20], and retail food sources [21]. Increasing evidences have suggested that gut phages also serve as a reservoir of ARGs in human [22], mice [23], and pigs [24–26]. Importantly, previous reports suggested that phages are involved in the acquisition, maintenance, and spread of ARGs [22]. Thus, characterizing the composition of ARGs in the gut phages is urgently needed and will facilitate the implementation of prevention strategies against spread of ARGs.

In this study, we used a toolbox for phage analysis (named PhaBOX) [27] to identify and characterize the gut phages in 112 pigs from seven pig breeds. The data indicated that a total of 174,897 non-redundant gut phage genomes were assembled and a total of 33,487 gut phage genomes were classified. We systematically analyzed the taxonomic classification, lifestyle prediction, host prediction, and functional annotation of gut phages in pigs. The data demonstrated that ARGs were also enriched in the gut phages and the most abundant antimicrobial resistance genotype was diaminopyrimidine resistance. These findings showed the communities and functions of gut phages and suggest that gut phages serve as a key reservoir of ARGs in pigs.

## Results

### Identification of gut phages in pigs from metagenomic data

**Fig. 1:**
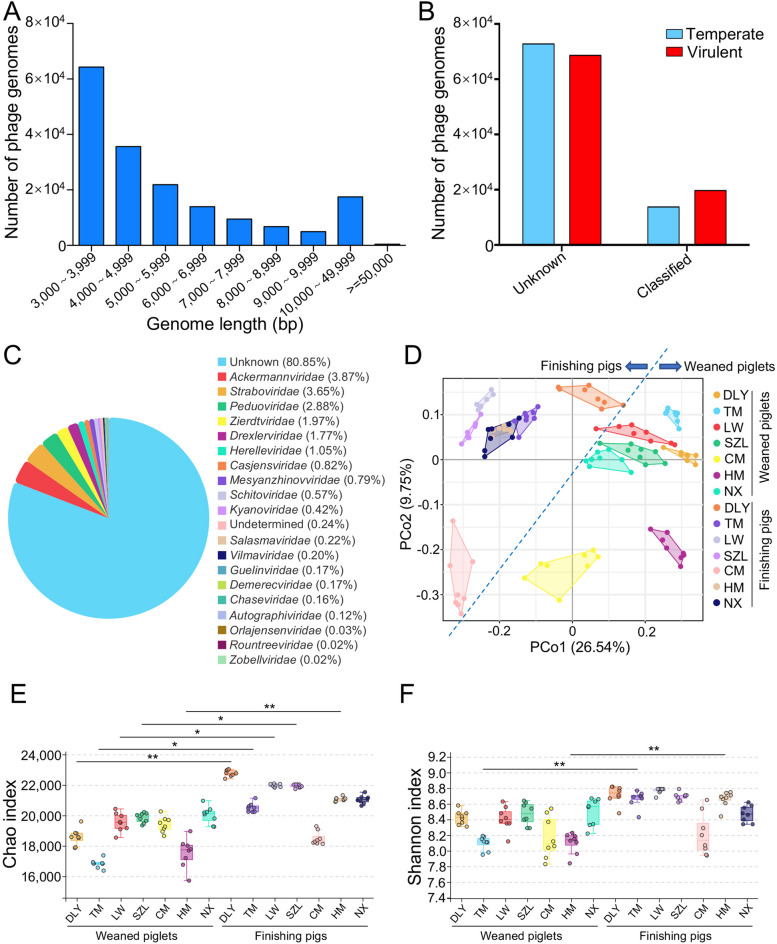
Identification of pig gut phages from metagenomic data and the diversity of gut phages. **A** Distribution of genome lengths in gut phage genomes. **B** Classification of gut temperate and virulent phages. **C** Proportion of taxonomic families identified in gut phage populations. **D** PCoA of gut phage communities based on the Bray–Curtis distance (DLY, Duroc × [Landrace × Yorkshire]; TM, Tibetan miniature; LW, Laiwu; SZL, Shaziling; CM, Congjiang miniature; HM, Huanjiang miniature; NX, Ningxiang). **E** Chao index analysis of gut phage communities. **F** Shannon index analysis of gut phage communities. Data are presented as mean ± SEM (*n* = 8) and evaluated by Kruskal–Wallis test in **E** and **F**. **p < 0.01, *p < 0.05

To investigate the communities and functions of gut phages in 112 pigs (including 56 weaned piglets and 56 finishing pigs) from seven pig breeds, including commercial Duroc × [Landrace × Yorkshire] (DLY), Chinese native Tibetan miniature (TM), Chinese native Laiwu (LW), Chinese native Shaziling (SZL), Chinese native Congjiang miniature (CM), Chinese native Huanjiang miniature (HM), and Chinese native Ningxiang (NX), we used the PhaBOX strategy to assemble the gut phage genomes from metagenomic data (Fig. S1). A total of 174,897 non-redundant gut phage genomes were assembled from 112 metagenomes and the assembled genome length mainly ranged from 3000 to 7000 bp (Fig. 1A). After taxonomic classification, a total of 33,487 gut phage genomes were classified, whereas a total of 141,410 gut phage genomes were not found in existing databases, suggesting the potential of exploring gut phages in pigs (Fig. 1B). We used the PhaTYP tool in PhaBOX to predict the lifestyle of phages and the results showed that the number of classified virulent phage genomes was larger than that of classified temperate phage genomes (Fig. 1B). These classified gut phage genomes mainly belonged to phage families such as *Ackermannviridae*, *Straboviridae*, *Peduoviridae*, *Zierdtviridae*, *Drexlerviridae*, and *Herelleviridae*, and all these phages belonged to class *Caudoviricetes*, phylum *Uroviricota* (Fig. 1C).

### Diversity and taxonomic composition of gut phages in pigs

We investigated the diversity of gut phage populations. The results of principal coordinates analysis (PCoA) showed the differences in the beta diversity amongst seven pig breeds, especially between commercial DLY pigs and Chinese native CM pigs (Fig. 1D). The data also suggested the differences in the beta diversity between weaned piglets that were aged 2 weeks after weaning and finishing pigs whose weights were close to the market weights, as evidenced by that most of weaned piglets clustered together and most of finishing pigs clustered together (Fig. 1D). The results showed that the Chao indexes in DLY, TM, LW, SZL, and HM finishing pigs were higher than those in corresponding weaned piglets, respectively (Fig. 1E). The Shannon indexes in TM and HM finishing pigs were higher than those in corresponding weaned piglets, respectively (Fig. 1F). Thus, these results revealed that the alpha diversity of gut phage community increased with the age of pig.

**Fig. 2:**
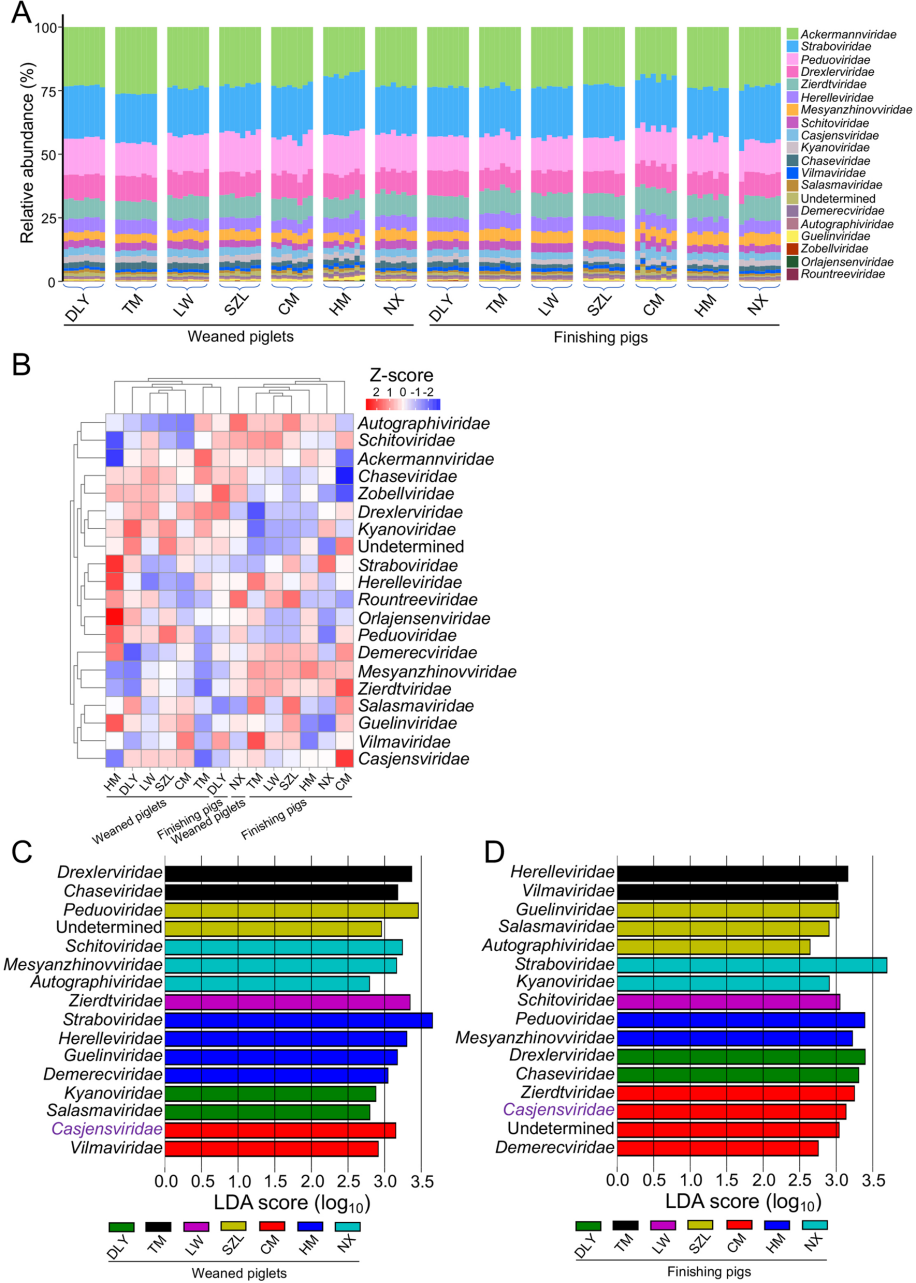
Analyses of the taxonomic composition of gut phages in pigs. **A** Taxonomic composition of gut phage populations at family level. **B** Heatmap analysis of gut phage populations at family level. **C** LEfSe analysis of gut phage populations in weaned piglets. **D** LEfSe analysis of gut phage populations in finishing pigs

We analyzed the taxonomic composition of gut phages in pigs. The stacked charts indicated that gut phages mainly belonged to phage families such as *Ackermannviridae*, *Straboviridae*, and *Peduoviridae*, followed by *Drexlerviridae*, *Zierdtviridae*, *Herelleviridae*, *Mesyanzhinovviridae*, *Schitoviridae*, and *Casjensviridae* (Fig. 2A). The gut phages in seven pig breeds exhibited distinct taxonomic compositions (Fig. 2A). The results of heatmap analysis showed most of weaned piglets clustered together and most of finishing pigs clustered together, suggesting an obvious distinction in taxonomic composition of gut phages between weaned piglets and finishing pigs (Fig. 2B). The Linear discriminant analysis Effect Size (LEfSe) of weaned piglets and finishing pigs showed the differences in taxonomic composition of gut phages amongst seven pig breeds (Fig. 2C, D). By comparing the results from LEfSe analysis, we found that the taxonomic composition of enriched phage families in gut phages of seven pig breeds were distinct between weaned piglets and finishing pigs (Fig. 2C, D). However, the family *Casjensviridae* was enriched in both CM weaned piglets and CM finishing pigs, suggesting that *Casjensviridae* may be a signature phage family in the gut phage populations of CM pigs (Fig. 2C, D).

### Characteristics of core, common, and unique gut phages in pigs

**Fig. 3:**
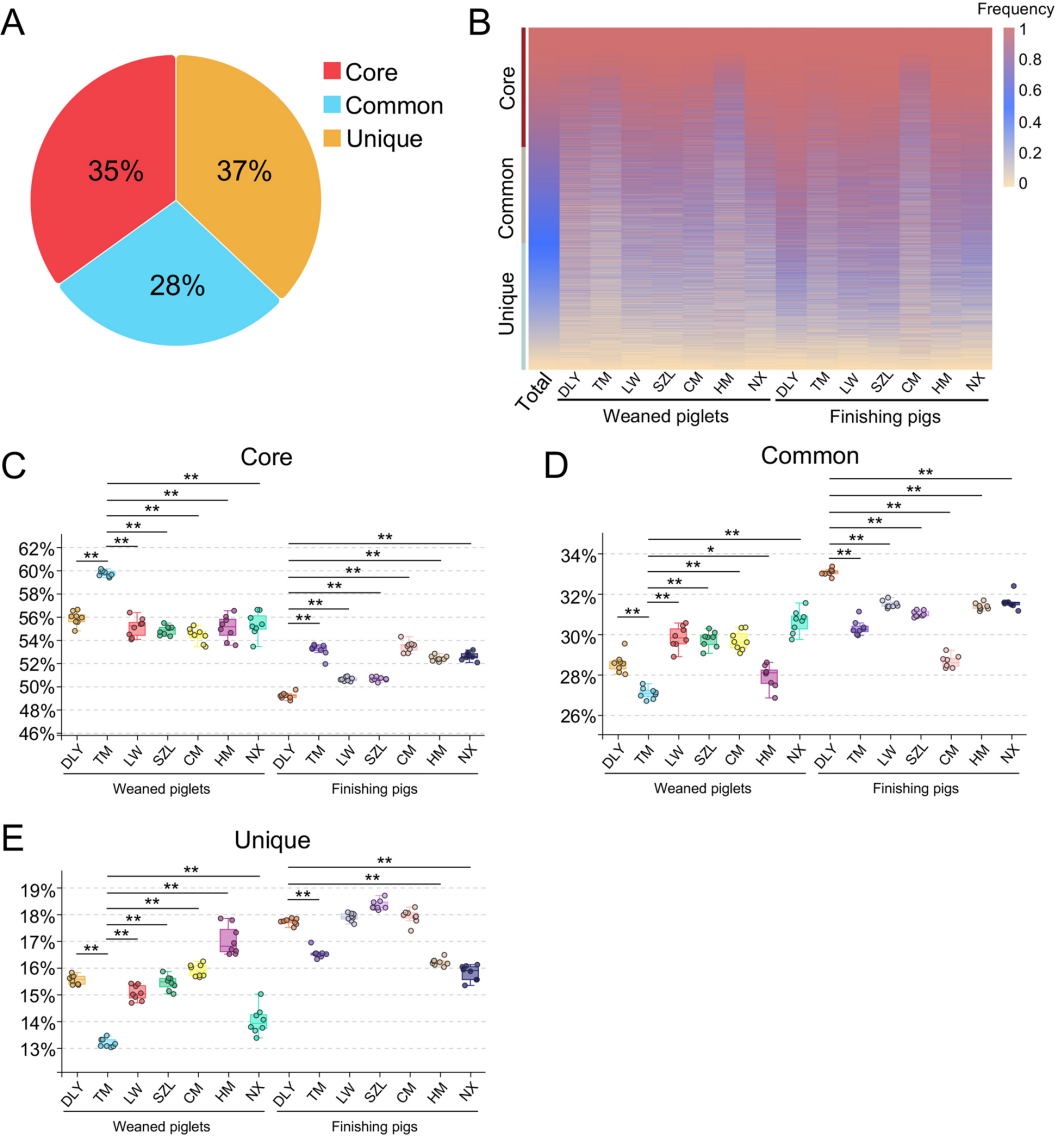
Analyses of core, common, and unique gut phages in pigs. **A** The proportion of core, common, and unique gut phages in the whole gut phage communities. **B** Heatmap analysis of core, common, and unique gut phages. The frequencies of phages are marked by different colors and each line is a phage. **C**–**E** Proportion of core (C), common (D), and unique (E) gut phages in seven pig breeds, respectively. Data are presented as mean ± SEM (*n* = 8) and evaluated by the one-way analysis of variance (ANOVA) test in **C**–**E**. **p < 0.01, *p < 0.05

To better evaluate the shared gut phageome of pigs, we analyzed the frequencies of gut phages in whole gut phage communities. The results showed that 35% of gut phages belonged to core gut phages that were shared by more than 80% of all pigs using similar criteria reported previously [2] (Fig. 3A). Twenty-eight percent of gut phages belonged to common gut phages that were shared by 50–80% of all pigs (Fig. 3A). Thirty-seven percent of gut phages belonged to unique gut phages that were shared by less than 50% of all pigs (Fig. 3A). The heatmap further showed the distribution in the frequencies of gut phages amongst seven pig breeds (Fig. 3B). The results indicated that the proportion of core gut phages in TM weaned piglets was the highest amongst the seven breeds of weaned piglets (Fig. 3C). The proportion of core gut phages in DLY finishing pigs was lowest amongst seven breeds of finishing pigs (Fig. 3C). However, the proportion of common gut phages in TM weaned piglets was lowest amongst seven breeds of weaned piglets (Fig. 3D). The proportion of common gut phages in DLY finishing pigs was the highest amongst the seven breeds of finishing pigs (Fig. 3D). The proportion of unique gut phages in TM weaned piglets was the lowest amongst the seven breeds of weaned piglets (Fig. 3E). The proportion of unique gut phages in DLY finishing pigs was higher than those in TM, HM, and NX finishing pigs (Fig. 3E). These findings suggested that Chinese native TM weaned piglets have the highest proportion of core gut phages amongst seven breeds of weaned piglets. The commercial DLY finishing pigs have the lowest proportion of core gut phages amongst seven breeds of finishing pigs.

### Comparison analyses of gut phages amongst pig breeds and the shifts in gut phages with the age of pig

**Fig. 4:**
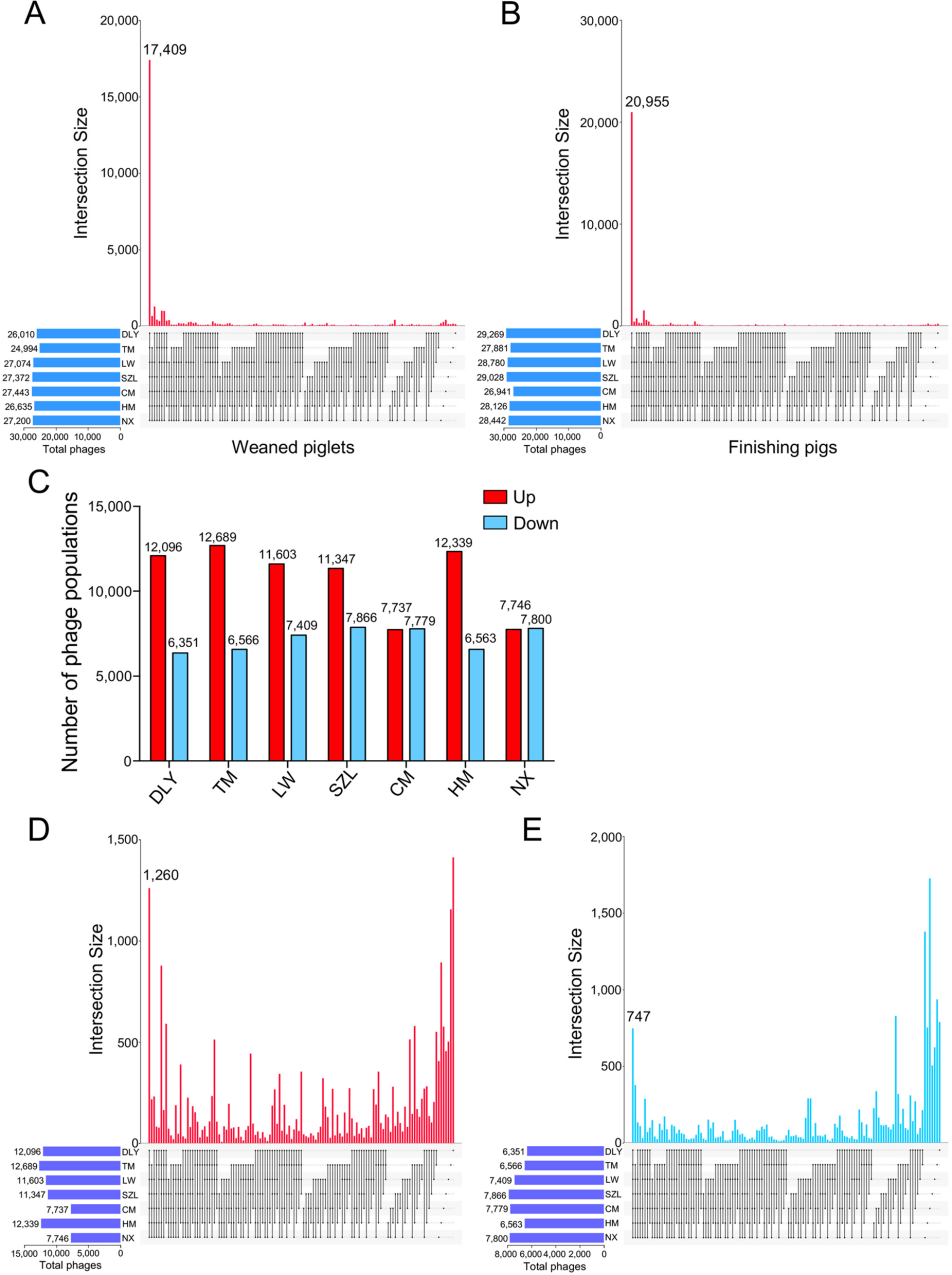
Comparative analysis of the gut phages amongst seven pig breeds and the shifts in the abundances of gut phages with the age of pig. **A** UpSet plot comparing the gut phage populations amongst seven breeds in weaned piglets. **B** UpSet plot comparing the gut phage populations amongst seven breeds in finishing pigs. **C** The number of gut phages whose abundances increased or decreased as the pigs aged. **D** UpSet plot comparing the gut phages whose abundances increased with the age of pig amongst seven breeds. **E** UpSet plot comparing the gut phages whose abundances decreased with the age of pig amongst seven breeds. Data are presented as mean ± SEM (*n* = 8) and evaluated by Wilcoxon rank sum test (**C**–**E**) and were shown in detail in Data S1

To evaluate the potential effects of breed and age on gut phages, we compared the compositions of gut phages amongst pig breeds and analyzed the shifts in gut phages with the age of pig. The results showed that a total of 17,409 common gut phages were identified in all seven breeds of weaned piglets (Fig. 4A). A total of 20,955 common gut phages were identified in all seven breeds of finishing pigs (Fig. 4B). These data suggested that most of the gut phages were shared by the seven pig breeds. Furthermore, the number of gut phage populations whose relative abundances increased with the age of pig (including DLY, TM, LW, SZL, and HM breeds) were larger than the number of gut phage populations whose relative abundances decreased with the age of pig (Fig. 4C). However, the number of gut phage populations whose relative abundances increased with the age of pig (including CM and NX breeds) were less than the number of gut phage populations whose relative abundances decreased with the age of pigs (Fig. 4C). A total of 1260 common gut phages whose relative abundances increased with the age of pig were identified in all the seven pig breeds (Fig. 4D). A total of 747 common gut phages whose relative abundances decreased with the age of pig were identified in all the seven pig breeds (Fig. 4E). However, a large proportion of unique gut phages whose relative abundances altered with the age of pig were identified in seven pig breeds, respectively (Fig. 4D, E). Together, these findings suggested that most of gut phages were shared by the seven pig breeds, whereas the shifts in the relative abundances of gut phages with the age of pig were different amongst pig breeds.

### Host prediction for the gut phages in pigs

**Fig. 5:**
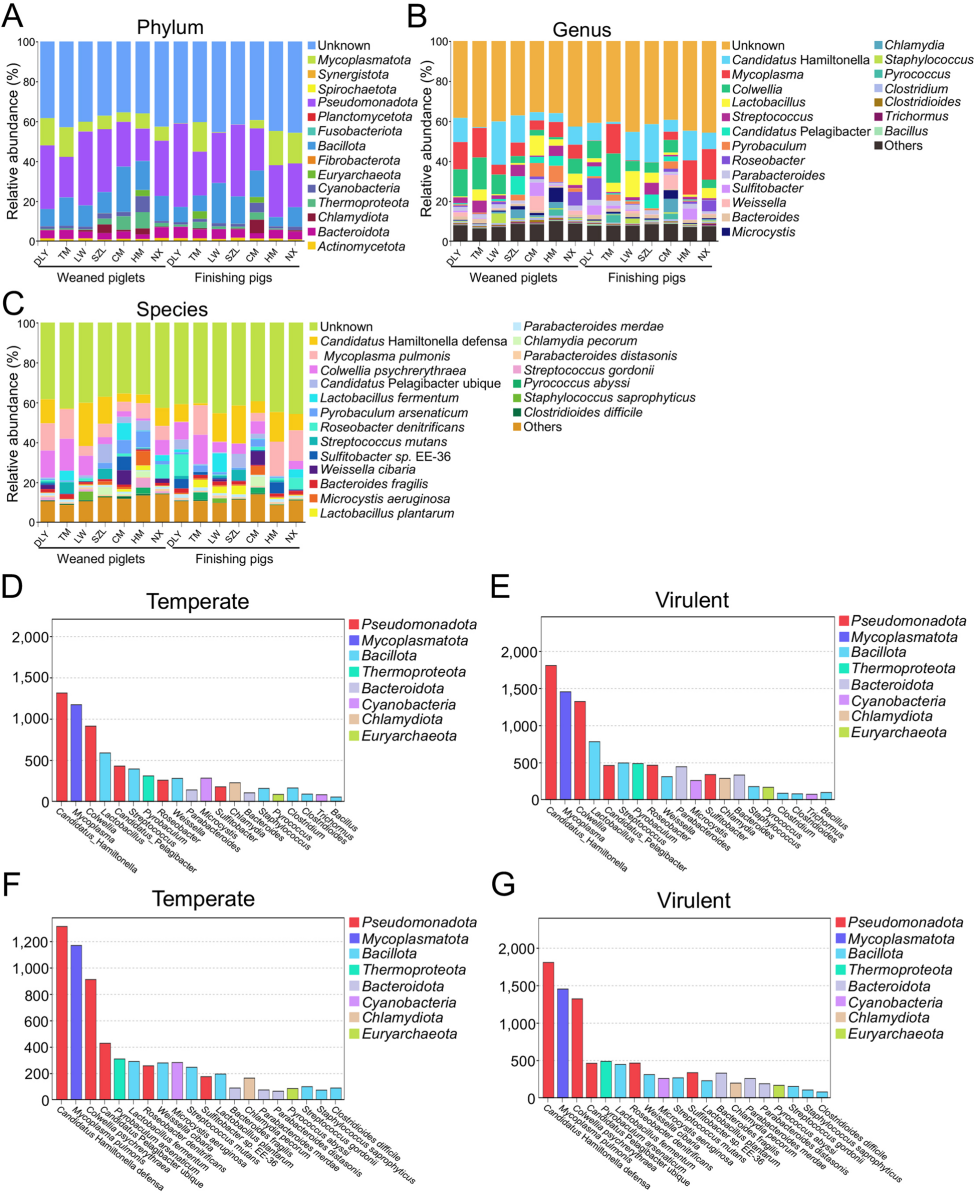
Host prediction analysis of gut phages in pigs. The taxonomic compositions after host prediction at phylum (**A**), genus (**B**), and species (**C**) levels, respectively. The number of temperate (**D**) and virulent (**E**) phage populations associated with host genera, respectively. The number of temperate (**F**) and virulent (**G**) phage populations associated with host species, respectively

Given that host prediction contributes to analyzing the potential functions of gut phages in gut microbial communities, we predicted the bacterial hosts of the gut phages identified using the CHERRY tool in PhaBOX. The results showed that these gut phages were predicted to infect a broad range of 14 phyla of prokaryotes, such as *Bacteroidota*, *Bacillota*, *Pseudomonadota*, and *Mycoplasmatota* (Fig. 5A). These gut phages were predicted to infect a broad range of 212 genera of prokaryotes, such as *Candidatus *Hamiltonella, *Mycoplasma*, *Colwellia*, and *Lactobacillus* (Fig. 5B). At species level, these gut phages were predicted to infect a broad range of 424 species of prokaryotes, such as *Candidatus *Hamiltonella defensa, *Mycoplasma pulmonis*, *Colwellia psychrerythraea*, *Candidatus *Pelagibacter ubique, and *Lactobacillus fermentum* (Fig. 5C). Interestingly, the results of lifestyle prediction of phages using PhaTYP tool in PhaBOX indicated that the number of virulent gut phage populations that infect hosts was larger than the number of temperate gut phage populations that infect the corresponding hosts at both genus (Fig. 5D, E) and species (Fig. 5F, G) levels.

### Broad metabolic functions are enriched in the gut phages in pigs

**Fig. 6:**
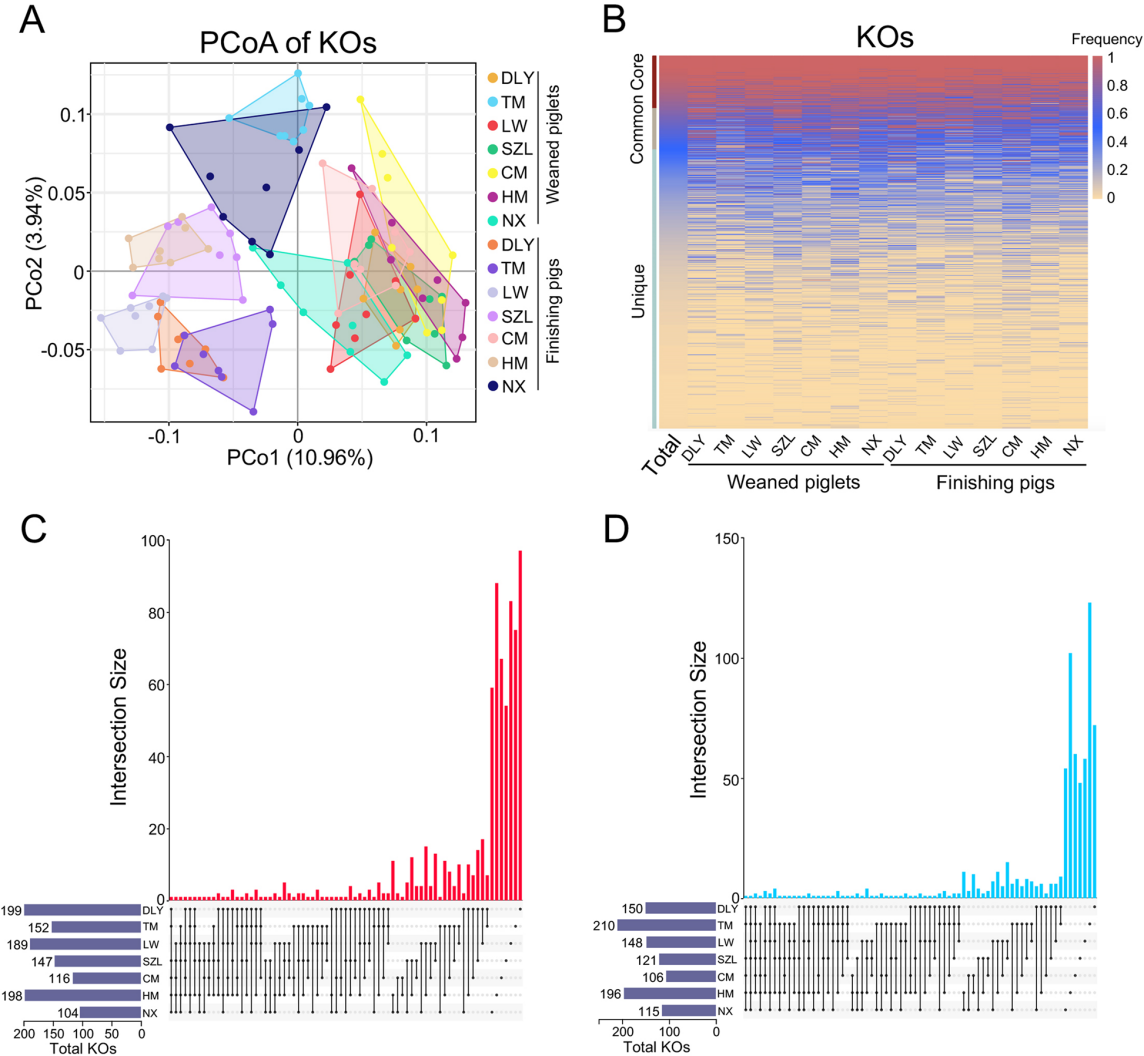
KEGG functions encoded in gut phages. **A** PCoA of KOs annotated in gut phages. **B** Heatmap analysis of core, common, and unique KOs. The frequencies of KOs are marked by different colors. **C** UpSet plot comparing the KOs whose abundances increased with the age of pig amongst seven breeds. **D** UpSet plot comparing the KOs whose abundances decreased with the age of pig amongst seven breeds. Data are presented as mean ± SEM (*n* = 8) and evaluated by Wilcoxon rank sum test (**C** and **D**) and were shown in detail in Data S2

To evaluate the potential metabolic functions enriched in gut phages in pigs, we annotated the phage-encoded genes using the Diamond software to align the phage genes against the Kyoto Encyclopedia of Genes and Genomes (KEGG) database. PCoA revealed a difference in the composition of KEGG orthologous groups (KOs) in gut phages amongst pig breeds and an obvious difference in the composition of KOs in gut phages between weaned piglets and finishing pigs (Fig. 6A). A total of 550 core KOs and 434 common KOs were identified in gut phages, respectively. Interestingly, a total of 2929 unique KOs were identified in gut phages (Fig. 6B). Furthermore, the UpSet plot analysis indicated that there is very few common KOs in gut phages that increased or decreased with the age of pig amongst seven pig breeds, suggesting a different shift in the KOs in gut phages with the age of pig amongst seven pig breeds (Fig. 6C, D).

**Fig. 7:**
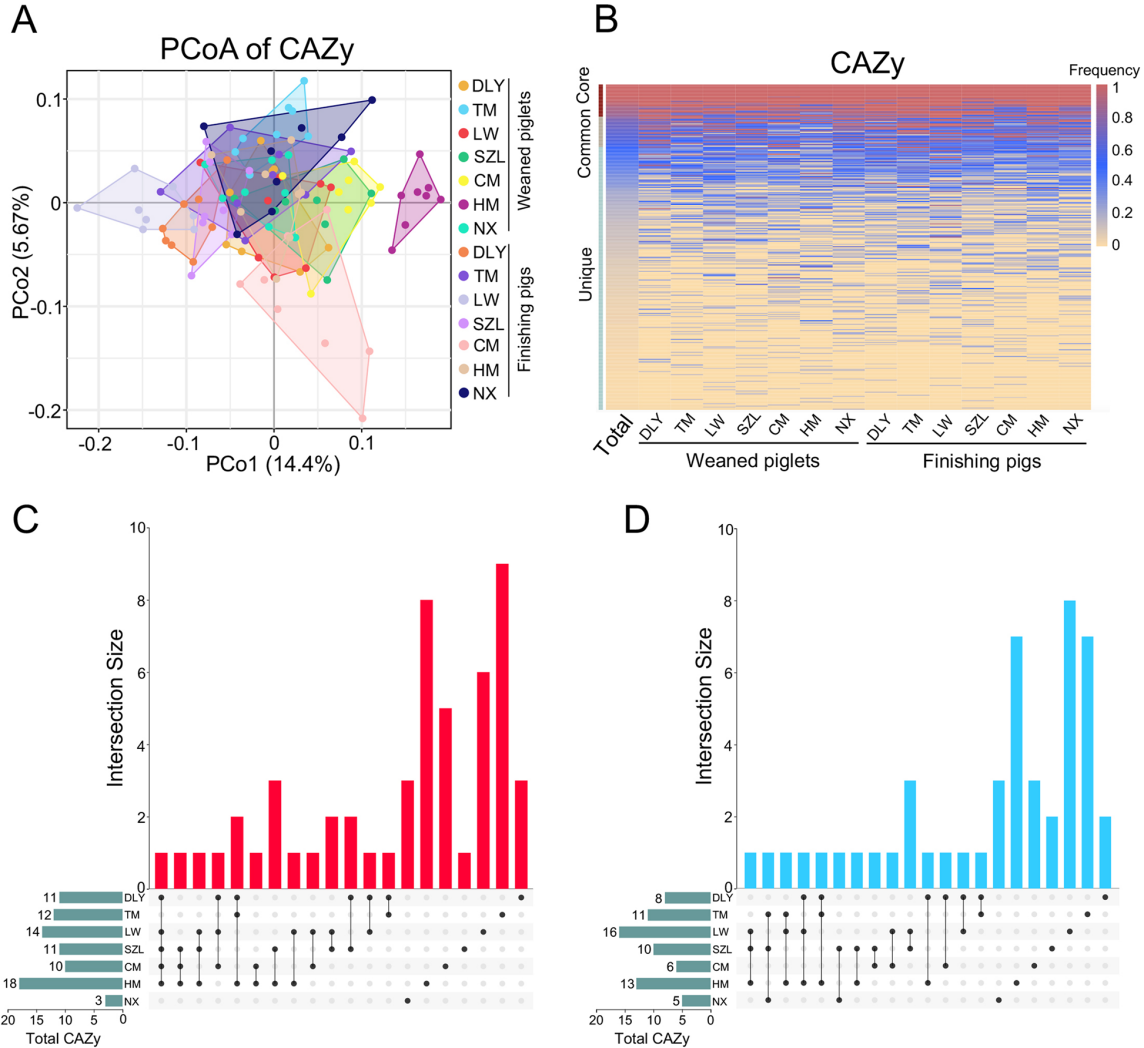
CAZy functions encoded in gut phages. **A** PCoA of CAZys annotated in gut phages. **B** Heatmap analysis of core, common, and unique CAZys. The frequencies of CAZys are marked by different colors. **C** UpSet plot comparing the CAZys whose abundances increased with the age of pig amongst seven breeds. **D** UpSet plot comparing the CAZys whose abundances decreased with the age of pig amongst seven breeds. Data are presented as mean ± SEM (*n* = 8) and evaluated by the Wilcoxon rank sum test (**C** and **D**) and were shown in detail in Data S3

Considering that the phages may be involved in carbon metabolism in animals and environments [28–30], we further annotated the phage-encoded genes using the dbCAN2 software against the carbohydrate-active enzymes (CAZy) database. PCoA did not show a difference in the composition of CAZys in gut phages amongst seven pig breeds (Fig. 7A). A total of 30 core CAZys were identified in gut phages, including 15 glycoside hydrolases (GHs), 8 glycosyltransferases (GTs), 5 carbohydrate esterases (CEs), 1 polysaccharide lyases (PLs), and 1 carbohydrate-binding modules (CBMs). A total of 28 common CAZys were identified in gut phages and a total of 244 unique CAZys were identified in gut phages (Fig. 7B). Furthermore, the UpSet plot analysis indicated that there is very few common CAZys in gut phages that increased or decreased with the age of pigs amongst seven pig breeds, suggesting a different shift in the CAZys in gut phages with the age of pig amongst seven pig breeds (Fig. 7C, D).

### Antimicrobial resistance is enriched in the gut phages in pigs

**Fig. 8:**
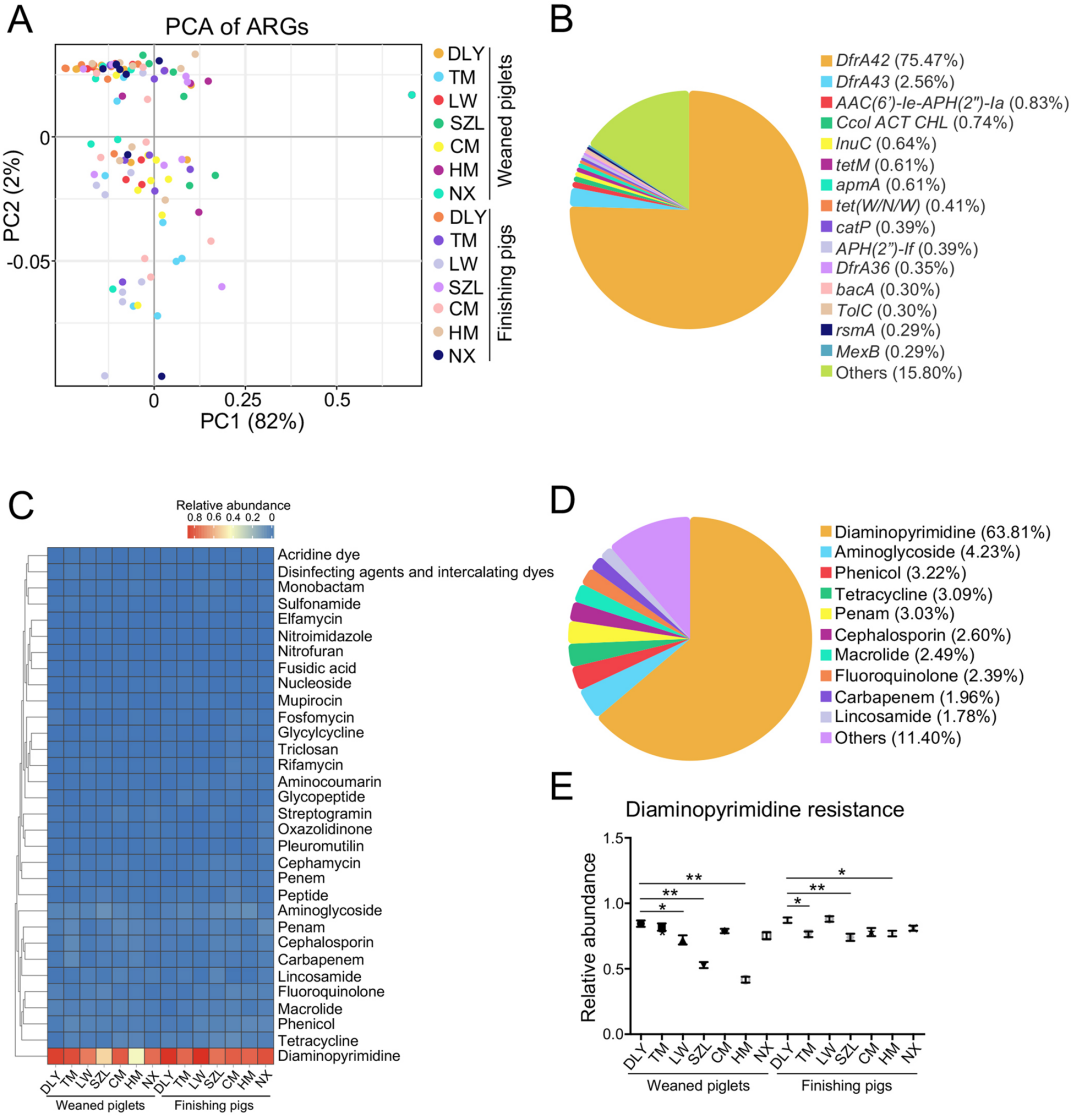
Identification of ARGs encoded in gut phages. **A** PCA of ARG profiles. **B** The mean proportion of ARGs in gut phages of 112 pigs. **C** Heatmap analysis of antimicrobial resistance types. **D** The mean proportion of antimicrobial resistance types in gut phages of 112 pigs. **E** The comparative analysis of diaminopyrimidine resistance type amongst seven breeds. Data are presented as mean ± SEM (*n* = 8) and evaluated by one-way ANOVA in **E**. **p < 0.01, *p < 0.05

Considering that phages have been recognized as a reservoir of ARGs [22], we investigated the composition of ARGs encoded in gut phages of pigs. The results of principal component analysis (PCA) did not show an obvious difference in the composition of ARGs amongst seven pig breeds (Fig. 8A). A total of 241 ARGs were identified in the gut phages of pigs. Of them, several ARGs (such as *DfrA42*, *DfrA43*, *AAC(6’)-Ie-APH(2″)-Ia*, *Ccol ACT CHL*, *lnuC*, *tetM*, *apmA*, and *tet(W/N/W)*) have a large average proportion and dominated in the ARGs of gut phages (Fig. 8B). Importantly, the most abundant ARG encoded in gut phages was the *DfrA42* that accounted for 75.47% of total ARGs, followed by *DfrA43* (Fig. 8B).

We also analyzed the antimicrobial resistance genotypes and obtained the genotype abundance profile in the gut phages. The data showed that a total of 32 antimicrobial resistance genotypes were classified (Fig. 8C) and several antimicrobial resistance genotypes (such as diaminopyrimidine, aminoglycoside, phenicol, tetracycline, penam, cephalosporin, macrolide, fluoroquinolone, carbapenem, and lincosamide) have a large average proportion in the antimicrobial resistance genotypes of gut phages (Fig. 8D). Of them, diaminopyrimidine resistance was the most abundant antimicrobial resistance genotype that accounted for 63.81% of total antimicrobial resistance genotypes (Fig. 8C, D). Interestingly, the relative abundances of diaminopyrimidine resistance in commercial DLY weaned piglets were higher than those in Chinese native LW, SZL, and HM weaned piglets (Fig. 8E). The relative abundances of diaminopyrimidine resistance in commercial DLY finishing pigs were higher than those in Chinese native TM, SZL, and HM finishing pigs (Fig. 8E). These findings suggested a larger spread risk of diaminopyrimidine resistance in gut phages in commercial DLY pigs than those in Chinese native pigs.

**Fig. 9:**
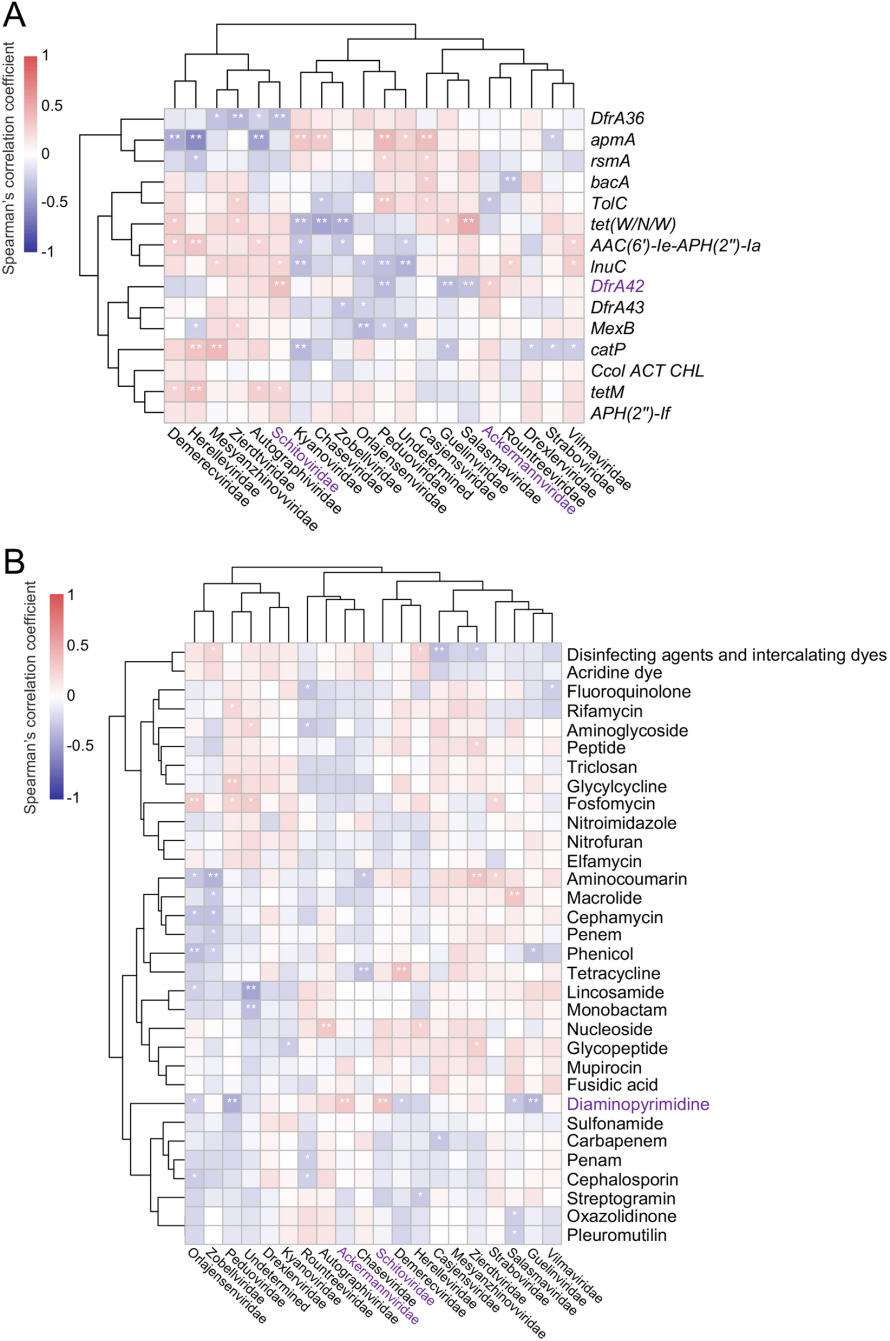
Correlation analysis of gut phages and antimicrobial resistance. **A** Heatmap for the Spearman’s correlation analysis of gut phages and ARGs (top 15 of mean proportion). **B** Heatmap for the Spearman’s correlation analysis of gut phages and antimicrobial resistance types. **p < 0.01, *p < 0.05

We performed the Spearman’s correlation analysis to analyze the potential gut phages associated with the ARGs. Heatmap showed that phage family *Casjensviridae* had obvious positive correlations with ARGs (including *apmA*, *rsmA*, *bacA*, and *TolC*) in gut phages (Fig. 9A). Several phage families (such as *Zobellviridae*, *Orlajensenviridae*, *Drexlerviridae*, and *Straboviridae*) had obvious negative correlations with ARGs in gut phages (Fig. 9A). Interestingly, two phage families (*Schitoviridae* and *Ackermannviridae*) had obvious positive correlations with *DfrA42*, the most abundant ARG in gut phages (Fig. 9A). We also performed a Spearman’s correlation analysis to analyze the potential gut phages associated with the antimicrobial resistance genotypes. The results revealed that two phage families (*Schitoviridae* and *Ackermannviridae*) had obvious positive correlations with diaminopyrimidine resistance, the most abundant antimicrobial resistance genotypes in gut phages (Fig. 9B). The phage family *Demerecviridae* had an obvious positive correlation with tetracycline resistance (Fig. 9B). The phage family *Salasmaviridae* had an obvious positive correlation with macrolide resistance (Fig. 9B). Together, these findings suggested that gut phages also carried ARGs and the most abundant antimicrobial resistance genotype was the diaminopyrimidine resistance.

## Discussion

Given that most gut phages are difficult to culture, metagenomic analysis is widely used to evaluate the compositions and functions of these phages [2, 31, 32]. Compared to the viral metagenomes, whole microbial communities (bulk) metagenomes have several advantages, including capturing the sequences of both extracellular and intracellular viruses, and not being influenced by the biases induced by whole-genome amplification [33]. However, detecting the phage contigs from metagenomes accurately is a challenge due to the limited reference genomes and high diversity of communities [3]. Recently, the PhaBOX was developed for phage contig identification, lifestyle prediction, taxonomic classification, and host prediction from the bulk metagenomes conveniently [27]. The PhaBOX strategy comprises four tools, including PhaMer for phage identification [34], PhaTYP for lifestyle prediction [35], PhaGCN for taxonomy classification [36], and CHERRY for host prediction [37]. These tools combine the strength of alignment-based strategies and deep learning models to learn different sequence-based features [27] and have been used in recent studies [38–44]. In our study, a total of 174,897 non-redundant gut phage genomes were assembled and a total of 33,487 gut phage genomes were classified from 112 pig gut metagenomes using the PhaBOX strategy. We also systematically analyzed the taxonomic classification, lifestyle prediction, and host prediction of gut phages in pigs, respectively. Thus, combining the metagenomics sequencing and PhaBOX strategy contributes to comprehensively revealing the composition and function of gut phages.

Most previous studies have focused on the gut phageome of weaned piglets and diarrhea piglets [45–48]. However, little is known on the gut phageome of finishing pigs. Our research first explored the phage landscape of both weaned piglets and finishing pigs across seven pig breeds and found that the alpha diversity of gut phages was increased with the age of pigs, consisting with previous studies on gut bacterial and fungal communities in pigs [49, 50]. These results filled a gap for the gut phageome of finishing pigs. An obvious distinction in taxonomic composition of gut phages between weaned piglets and finishing pigs may be caused by different age and diet, as previously mentioned [51, 52]. A healthy gut phageome comprising both core and common phages is necessary for maintaining gut microbiome function and thereby contributes significantly to host health [2, 46]. Our results showed that 35% of gut phages were core gut phages that were shared by more than 80% of all pigs and the 28% of gut phages were common gut phages that were shared by 50–80% of all pigs. These core and common phages in pigs used in our study may be important for pig health. Our data showed that the gut phages in seven pig breeds exhibited distinct communities and the gut phage communities changed with the age of pig. Furthermore, gut phages in these seven pig breeds mainly belonged to *Ackermannviridae*, *Straboviridae*, *Peduoviridae*, *Drexlerviridae*, *Zierdtviridae*, and *Herelleviridae*, different from the results from gut virome in other mammals [47, 48, 53]. These differences may be caused by different geography, diet, species, and breed, as previously mentioned [51, 52].

Gut phages may be integrated into the bacterial genome as lysogenic prophages [54, 55]. Predicting the host of phages and comprehending the interactions between phages and bacteria are important to elucidating the function of phages. Here, we revealed that gut phages in pigs were predicted to infect a broad range of 14 phyla of prokaryotes, such as *Bacteroidota*, *Bacillota*, *Pseudomonadota*, and *Actinomycetota*, that were consistent with previous studies on the neonatal piglets [48] and weaned pigs [47]. Previous studies showed that gut phages in pigs were predicted to infect several genera of prokaryotes, such as *Clostridium*, *Lactobacillus*, *Bacillus*, *Streptococcus*, and *Bacteroides* [25, 52, 56, 57], that were also consistent with our data. However, previous studies rarely revealed the host species level-taxonomic composition of gut phages in pigs. Viruses are reported to be widely involved in carbon metabolism in animals and environments [28–30]. To explore the potential functions of gut phage in these seven pig breeds, we annotated the phage-encoded genes using the CAZy database. A total of 30 core CAZys, including GHs, GTs, CEs, PLs, and CBMs, were identified in gut phages. Consistently, the GHs, GTs, CEs, and CBMs in pig gut virome were also identified in previous studies [45, 48, 52]. These results suggested that the gut bacteria which acquire these CAZys from the gut phage reservoir may gain additional foraging capacity and thus CAZys in phages may confer important roles in the gut microbial ecosystem.

The transfer of ARGs in the environment is a threat to both human and animal health [15]. Intensive pig farms are hotspots for ARGs transmission because ARGs are frequently found in pig gut microbiome [58–60]. Besides the bacteria, phages also carried ARGs in several environments, such as fresh water [17], marine habitats [18], raw sewage [19, 20], and retail food sources [21]. Gut phages also serve as a reservoir of ARGs in human [22], mice [23], and pigs [24–26]. However, recent studies on pig gut microbiome reported that the phages rarely carried the ARGs [52, 61]. In the present study, we identified a total of 241 ARGs in gut phages in pigs. Importantly, the most abundant ARGs encoded in gut phages across seven pig breeds were the *DfrA42* that accounted for 75.47% of total ARGs, followed by *DfrA4*. The *DfrA42* and *DfrA43* are novel genes identified from trimethoprim-resistant *Proteus* strains and confer diaminopyrimidine resistance [62]. Our data also suggested a larger spread risk of diaminopyrimidine resistance in gut phages in commercial DLY pigs than those in Chinese native pigs. Considering that phages are involved in the acquisition, maintenance, and spread of ARGs [22], our data will facilitate the implementation of prevention strategies against spread of ARGs in pig production.

## Conclusions

In summation, we delineate the landscape for gut phages in both weaned piglets and finishing pigs across seven pig breeds. The taxonomic classification, lifestyle prediction, host prediction, and functional annotation of gut phages are systematically analyzed. Our data also suggest that gut phages serve as a key reservoir of ARGs in pigs. These findings facilitate a panoramic view of the pig gut phageome and provide insights into the functional contributions of gut phages in pigs.

## Methods

### Collection of fecal samples from pigs and extraction of microbial genomic DNA

A total of 112 fecal samples were individually collected from 56 weaned piglets and 56 finishing pigs from seven pig breeds, including DLY, TM, LW, SZL, CM, HM, and NX (*n* = 8). The detailed information for these pigs was described in our previous study [49]. We used the combined method of CTAB and beat-beading to extract the microbial genomic DNA from feces of pigs. The procedures for DNA extraction were described previously in detail [49].

### Metagenomic sequencing and data analysis

Illumina TruSeq DNA PCR-Free Library Preparation Kit (Illumina, USA) was used to prepare the metagenomic libraries. The qualified libraries were sequenced on a HiSeq X Ten System (Illumina) through the 150-bp paired-end strategy. The SOAPnuke software (v1.5.6) was used to remove the low-quality, duplicate, and adapter contamination reads from the raw sequencing data and then generate the high-quality sequencing reads. The Bowtie2 software (v2.2.5) was used to trim the host genomic reads and obtain the final clean data. Then, we used the IDBA-UD software (v1.1.3) to de novo assemble the high-quality clean reads for generating metagenomic contigs individually.

### Phage identification, taxonomic classification, lifestyle prediction, and host prediction from metagenomic data

The PhaBOX strategy integrates several tools, including PhaMer, PhaTYP, PhaGCN, and CHERRY, for phage identification, lifestyle prediction, taxonomy classification, and host prediction, respectively. We used the PhaBOX strategy to identify and characterize the gut phages from the metagenomic assembled contigs according to the default parameters [27]. The contigs predicted as candidate phage genomes from each sample were merged and clustered using the CD-Hit software (v4.6) based on the criteria of identity of > 95% and coverage of > 90% to remove redundant genomes. To estimate the phage abundance, the Bowtie2 software (v2.2.5) was used to align the clean reads to non-redundant phage genome clusters. The obtained the phage genome abundance profile was then quantified by the Salmon software (v0.9.1). Alpha diversity indexes, including Chao index and Shannon index, were calculated by the USEAECH software (v10.0.240). The scatter diagram of PCoA based on the Bray–Curtis distance was conducted using OmicShare. Heatmaps and histograms for taxonomic compositions were generated using R software (v3.4.1). LEfSe analysis was performed by LEfSe software (v1.0) to classify the significant different abundant biomarkers. The UpSet plots for phages amongst different groups were conducted by OmicShare.

### Analyses of KEGG and CAZy functions encoded in phages

The predicted genes of phages from each sample were subjected to functional analysis. Diamond software (v0.8.23.85) was used to align the phage genes against the KEGG database (v93.0) and the dbCAN2 software (v7.0) was used for the CAZyme database annotation. The functional abundance profiles were generated by summarizing the gene abundance profile. PCoA for KOs and CAZys based on the Bray–Curtis distance was conducted using OmicShare. Heatmaps for KOs and CAZys were generated using the R software. UpSet plots for KOs and CAZys were conducted by OmicShare.

### Identification of ARGs and ARG-phage correlation analysis

The RGI software (v5.2.0) was used to identify the ARGs against the CARD databases. The obtained ARG abundance profile was generated by summarizing the gene abundance profile. The PCA for ARGs was conducted using the OmicShare. The heatmap for antimicrobial resistance genotypes was generated using R software. Spearman’s correlation analysis between the gut phage abundance and ARGs abundance was conducted using R software. Spearman’s correlation analysis between gut phage abundance and antimicrobial resistance genotypes abundance was conducted using R software.

### Statistical analysis

Both GraphPad Prism (v6.0c) and R (v3.4.1) software were used for statistical analyses. The Kruskal–Wallis test, Wilcoxon rank sum test, one-way analysis of variance, and Spearman’s correlation test were conducted. Statistical significance was set at *p* < 0.05.

Supplementary information.

### Supplementary Information

**Additional file 1:** Fig. S1 Workflow for analyzing the pig gut phageome from metagenomic data (DLY, Duroc × [Landrace × Yorkshire]; TM, Tibetan miniature; LW, Laiwu; SZL, Shaziling; CM, Congjiang miniature; HM, Huanjiang miniature; NX, Ningxiang)

**Additional file 2:** Data S1 Detailed information for comparing the abundances of gut phages between weaned piglets and finishing pigs

**Additional file 3:** Data S2 Detailed information for comparing the abundances of KOs between weaned piglets and finishing pigs

**Additional file 4:** Data S3 Detailed information for comparing the abundances of CAZys between weaned piglets and finishing pigs

## Data Availability

Raw sequencing data were deposited in China National GeneBank Sequence Archive of the China National GeneBank DataBase with accession number CNP0002106.
