# Supplementary figures and images for: Characterizing the gut phageome and phage-borne antimicrobial resistance genes in pigs
Source: Microbiome. 2024 Jun 5;12:102. doi: 10.1186/s40168-024-01818-9 (PMC11151549; doi:10.1186/s40168-024-01818-9)

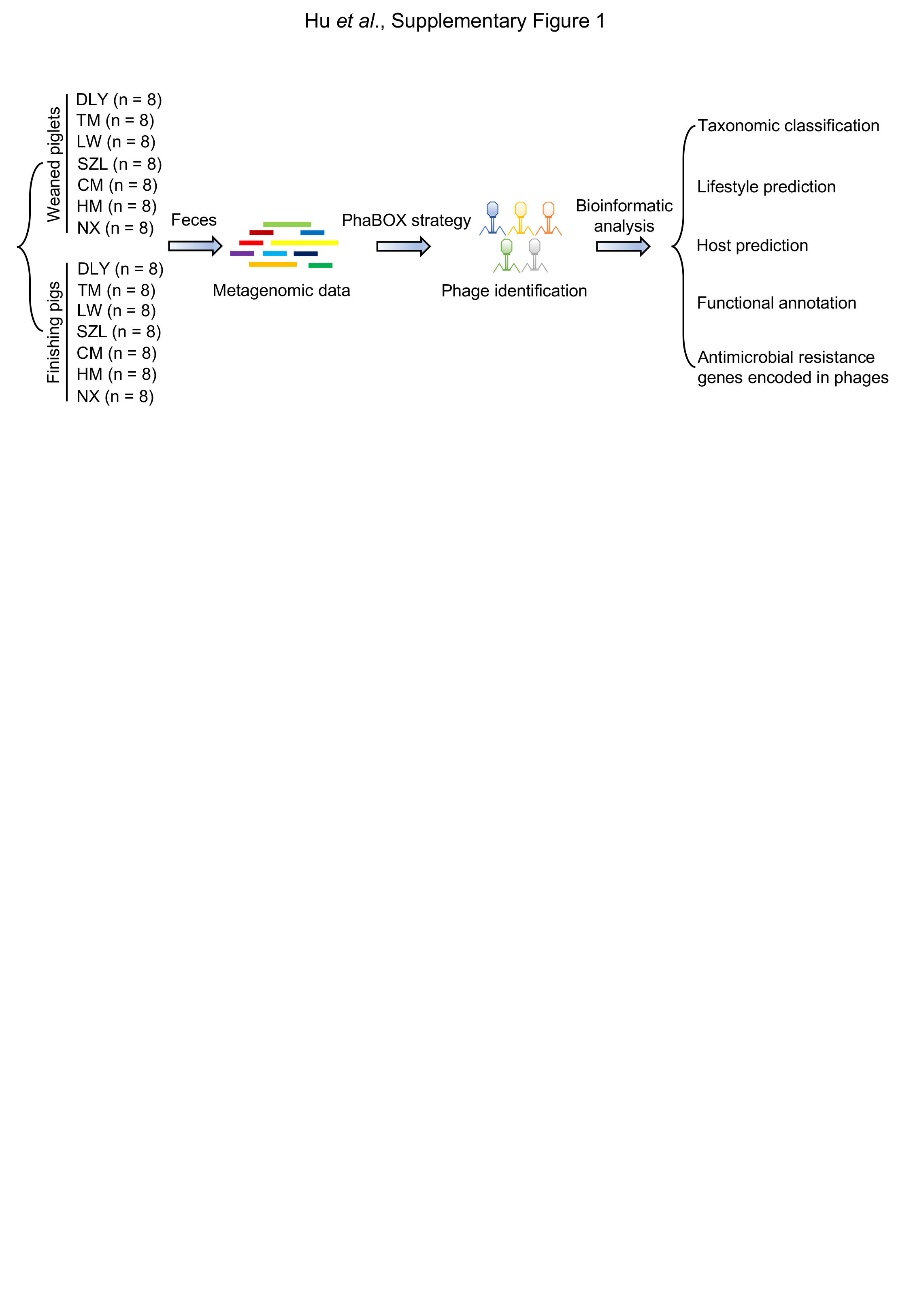

Supplement: Supplementary file 5 — Additional file 4: Data S3 Detailed information for comparing the abundances of CAZys between weaned piglets and finishing pigs [file 40168_2024_1818_MOESM4_ESM.jpg]
